# Association of Ureaplasma infection pattern and azithromycin treatment effect with bronchopulmonary dysplasia in Ureaplasma positive infants: a cohort study

**DOI:** 10.1186/s12890-023-02522-4

**Published:** 2023-06-26

**Authors:** Xueyu Chen, Xuemei Huang, Yanqing Lin, Bingchun Lin, Chunyu Yang, Zhifeng Huang, Chuanzhong Yang

**Affiliations:** 1 Department of Neonatology, The First School of Clinical Medicine, Shenzhen Maternity & Child Healthcare Hospital, Southern Medical University, Shenzhen, China; 2 Department of Neonatology, Liuzhou Maternity and Child Healthcare Hospital, Affiliated Maternity Hospital and Affiliated Children’s Hospital of Guangxi University of Science and Technology, Guangxi, China; 3 Department of Radiology, The First School of Clinical Medicine, Shenzhen Maternity & Child Healthcare Hospital, Southern Medical University, Guangzhou, China

**Keywords:** Chest X-ray, Interstitial pneumonia, Lung development

## Abstract

**Background:**

It is unclear whether Ureaplasma-associated pneumonia and azithromycin treatment affect the risk for bronchopulmonary dysplasia (BPD).

**Methods:**

A retrospective cohort study was performed in very low birth weight (VLBW) infants who tested positive for Ureaplasma within 72 h after birth in a tertiary unit. Chest X-ray (CXR) and laboratory test were performed before and after azithromycin treatment. Multivariate logistic regression analysis was used to identify the independent association between BPD and Ureaplasma-associated pneumonia, as well as BPD and effective azithromycin treatment.

**Results:**

A total of 118 infants were included in the current study, of whom 36 developed BPD (defined as supplemental oxygen needed at postmenstrual age 36 weeks or discharge). The rate of BPD was significantly higher in infants with Ureaplasma-associated pneumonia (44.6%) compared to infants with Ureaplasma colonization (17.7%, *P* = 0.002). After adjusting for confounders, an effective azithromycin treatment was significantly associated with reduced risk of BPD [odd ratio (OR) 0.011; 95% confidence interval (CI): 0.000–0.250), whereas Ureaplasma-associated pneumonia was not significantly associated with BPD (OR 1.835; 95% CI: 0.548–6.147).

**Conclusion:**

Effective Azithromycin treatment in Ureaplasma positive VLBW infants was associated with a reduced risk of BPD.

**Supplementary Information:**

The online version contains supplementary material available at 10.1186/s12890-023-02522-4.

## Background

Bronchopulmonary dysplasia (BPD) is a common complication in premature infants and remains prevalent in extremely preterm infants despite great advances in perinatal care [1, 2]. The pathogenesis of BPD is unclear and multifactorial, including but not restricted to oxygen toxicity, mechanical ventilation-induced lung injury and infection/inflammation [3]. Ureaplasma spp. has been reported as one of the earliest microbiotas colonized in the airway of premature infants, potentially contributing to the inflammatory and fibrotic profile of BPD [4]. Ureaplasma spp. has two species, *Ureaplasma parvum* and *Ureaplasma urealyticum*, with 14 known serovars. Both species were linked with preterm premature rupture of membranes (PPROM), chorioamnionitis and preterm labor. The association between Ureaplasma and BPD was first discussed by Holtzman et al. in 1989, who concluded that there was not enough evidence to prove the association [5]. Several studies also investigated the association between Ureaplasma and BPD, yielding conflicting findings [6–9]. Castro-Alcaraz and colleagues found the pattern of Ureaplasma colonization may be related to BPD, given that infants with persistent Ureaplasma had an increased risk of BPD [10]. In a systematic review and meta-analysis, pulmonary colonization with Ureaplasma increased the risk of BPD by three folds in premature infants [11]. However, a recent randomized trial reported that the eradication of Ureaplasma by azithromycin failed to decrease the risk of BPD [12]. Although the trial was underpowered to detect a difference in BPD due to funding problems [13], it indirectly supports the idea that Ureaplasma was not associated with BPD [14]. Noticeably, the detection and diagnosis criteria of Ureaplasma and BPD vary greatly among these studies, which may partially explain the elusive association between Ureaplasma and BPD.

In clinical practice, we observed that infants at a radiographically infectious status display a different respiratory clinical course than those with colonization alone. Inspired by Castro-Alcaraz and colleagues who found the pattern of Ureaplasma colonization was essential [10], this study aims to investigate the association between the proven infection of Ureaplasma with BPD and to evaluate the effect of azithromycin treatment on BPD.

## Materials and methods

### Study design

A retrospective cohort study was conducted at a level three neonatal intensive care unit (NICU), from June 2017 and October 2019. During the study period, preterm infants with a birth weight less thn 1500 g were routinely screened for Ureaplasma within 12 h after admission using a commercial kit (Rendu Biotechnology, Shanghai, China) by Real-time polymerase chain reaction (RT-PCR) in nasopharyngeal swabs. The RT-PCR assay detects both U. urealyticum and U. parvum, and Ureaplasma was used in the following text. The study was conducted in accordance with the Declaration of Helsinki and approved by the institute Ethical Committee. Written informed consent from the participants’ legal guardian/next of kin was not required in this study in accordance with the national legislation and the institutional requirements.

### Definition

The primary outcome is bronchopulmonary dysplasia (BPD), diagnosed as an oxygen requirement at 36 weeks’ postmenstrual age (PMA) or discharge [15]. Secondary outcomes included hospital death, retinopathy of prematurity (ROP), duration of invasive and non-invasive ventilation, duration of supplemental oxygen and hospitalization. Chest-X-ray (CXR) score, assessed by the total score of three aspects: diffuse granularity, interstitial changes, and emphysema, was used to assess the pulmonary infection status in Ureaplasma positive VLBW infants [16–18] (Table 1). Ureaplasma-associated pneumonia was defined as Ureaplasma Rt-PCR positive with clinical manifestation of CXR score ≥ 2 (Table 1) and an increased leukocyte ≥ 25*10^9^/L. Ureaplasma colonization was defined as Ureaplasma Rt-PCR positive with no or mild CXR score <2, or leukocyte < 25*10^9^/L. Effect of azithromycin treatment was defined as improvement in both CXR (decrease in score) and laboratory leukocyte (less than 20*10^9^/L). The diagnostic criteria of Ureaplasma-associated pneumonia are summarized in Table 1. The CXR was assessed by one neonatologist and one radiologist, who was blinded to the study design. The representative photos of CXR were displayed in the Supplemental figure.

**Table 1:** Algorithm for Ureaplasma associated pneumonia

RT-PCR assay	Chest radiography			Leukocyte	Status
	Assessment	Degree-Score	Total score		
Ureaplasma positive	1.Diffuse granularity	None-0/ Mild-1/ Severe-2	≥ 2	≥ 25*10^9^/L	Ureaplasma associated pneumonia
	2.Interstitial changes				
	3.Emphysema		< 2	Any value	Colonization

Diffuse granularity, interstitial changes, and emphysema were assessed independently as none (score 0), mild (score 1), and severe (score 2). A total score was defined as the sum of scores from the three aspects. An infant with a total Chest X-Ray score of more than 2 and a leukocyte more than 25* 10^9^/L was defined as Ureaplasma-associated pneumonia otherwise was colonization. Be noted that this assessment was performed in infants tested Ureaplasma positive by RT-PCR

### Azithromycin treatment strategies

VLBW infants were routinely screened for Ureaplasma after admission. The testing result was available within 48–72 h. The treatment was initiated shortly after doctors received the results. There is no current census on azithromycin treatment for Ureaplasma in preterm infants. For infants with Ureaplasma-associated pneumonia, azithromycin was administered orally at a daily dose of ten mg/kg for a week followed by five mg/kg for the next week. This regimen was modified from Ballard et al., where they used ten mg/kg of azithromycin daily for seven days, followed by five mg/kg of azithromycin for an additional five weeks [19]. We shortened the course on consideration of the potential side effect of azithromycin. For infants with Ureaplasma colonization, ten mg/kg azithromycin was administered orally daily for three days, suspended for four days, then repeated for three days. Dosage of azithromycin for Ureaplasma colonization was modified from a local consensus [20]. Eleven infants in Ureaplasma colonized group completed half the treatment course (ten mg/Kg for three days) due to clinical sepsis and feeding intolerance. They were included in the analysis. No RT-PCR test for Ureaplasma was performed after azithromycin treatment. The efficacy of azithromycin treatment was thus assessed based on radiographic improvement and laboratory tests.

### Statistics

The sample size was calculated based on a pilot study, where the rate of BPD was 48% in Ureaplasma-associated pneumonic infants, and the rate was 20% in Ureaplasma colonized VLBW infants. At an 80% power and an *α* = 0.05, 42 infants in each group would be sufficient to detect a significant difference. All statistical analyses were performed with IBM SPSS Statistics 24.0. In the univariate analysis, the chi-square test was used to analyse categorical variables, the nonparametric McNemar test was used for comparison of paired categorical variables, and the Student t-test was used for normally distributed continuous variables. Data were reported as numbers (%), the mean ± standard deviation, and IQR, respectively. Variates with a *P*-value < 0.1 were included in the multivariate logistic analysis. Multivariate analysis was performed by binary logistic regression and odds ratios (OR) and 95% confidence intervals (CI) were calculated. Statistical significance was accepted at *P* < 0.05.

## Results

**Fig. 1:**
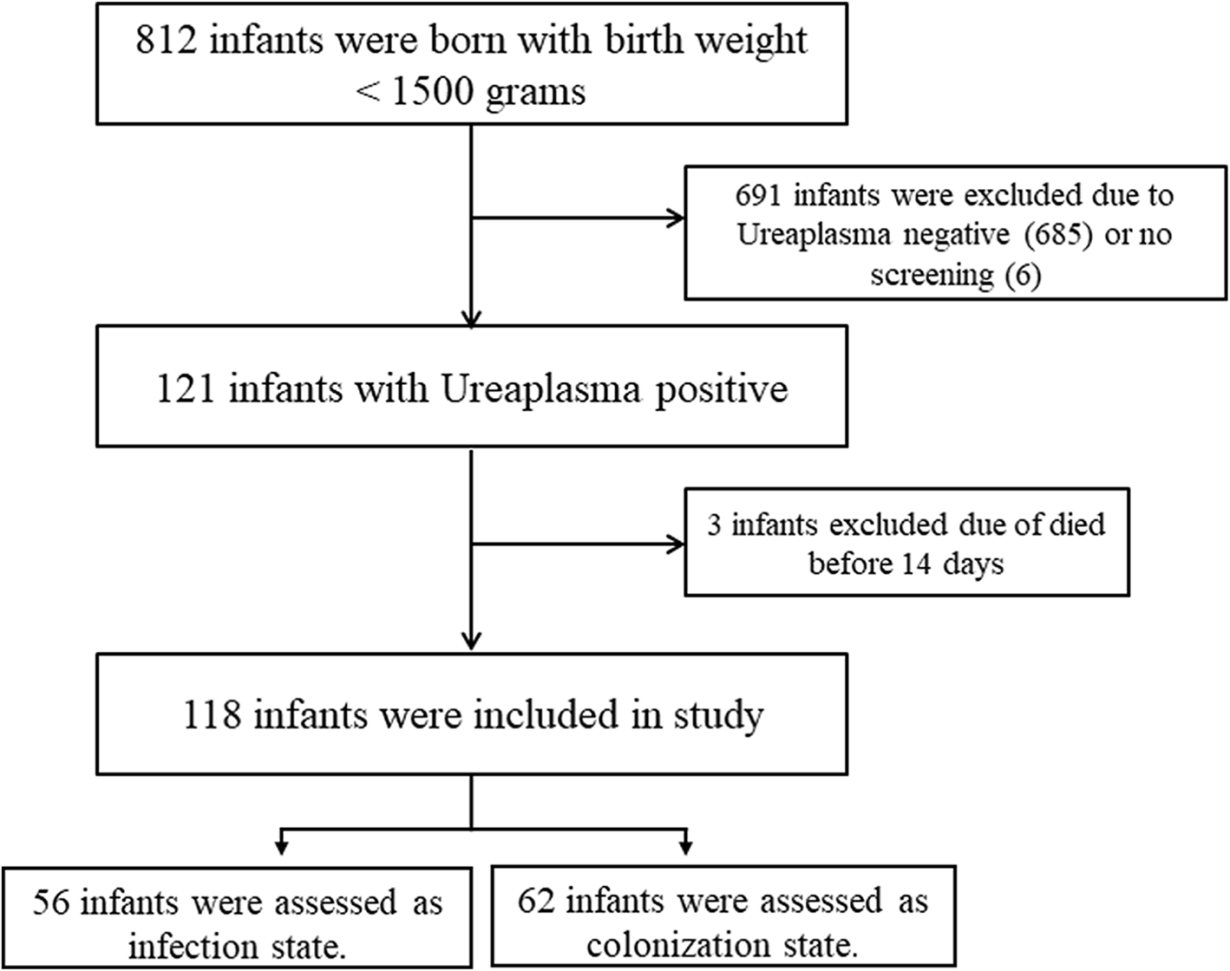
Flow chart of case selection

During the study period, 812 VLBW infants were admitted to the NICU. In these infants, 121 (14.9%) were tested positive for Ureaplasma. Three infants were excluded due to death before 14 days. A total of 118 infants are included in the final analysis (Fig. 1), of which 30.5% (36/118) developed BPD. The clinical characteristics of these infants were summarized in Table 2.

**Table 2:** Clinical characteristics stratified by Ureaplasma infection status

Characteristic	Ureaplasma associated pneumonia	Ureaplasma colonization	*p*-Value
	(*n* = 56)	(*n* = 62)	
Male, n (%)	30 (53.6)	42 (67.7)	0.115
GA, mean (SD)	27.2 (1.6)	28.8 (1.9)	0.000
BW, mean (SD)	1025 (257)	1233 (279)	0.000
Vaginal delivery, n (%)	38 (67.9)	37 (59.7)	0.357
1 min Apgar Score, median (IQR)	8.5 (4)	9 (2)	0.250
5 min Apgar Score, median (IQR)	10 (0)	10 (0)	0.011
Antenatal steroid, n (%)	42 (75.0)	54 (87.1)	0.092
NRDS (grade II and above)	17 (30.4)	7 (11.3)	0.010
SGA, n (%)	2 (3.6)	5 (8.1)	0.443
PPROM, n (%)	30 (53.6)	40 (64.5)	0.227
Surfactant, n (%)	41 (73.2)	24 (38.7)	0.000
Intubation, n (%)	29 (51.8)	18 (29.0)	0.012
EOS, n (%)	5 (8.9)	8 (12.9)	0.491

*Abbreviations*: *GA* Gestational age, *SD* Standard deviation, *BW* Birth weight, *IQR* Interquartile range, *NRDS* Neonatal respiratory distress syndrome, *SGA* Small for gestational age, *PPROM* Prelabour rupture of the membrane, *EOS* Early-onset sepsis, including suspected and culture-proven

Infants with Ureaplasma-associated pneumonia had significantly higher risk of BPD (44.6% vs. 17.7%, *P* = 0.002), longer invasive ventilation (1.0 vs 0.0 day, *P* = 0.004], longer non-invasive ventilation (37.0 vs 19.5 days, *P* < 0.001), longer oxygen support (58.0 vs 33.0 days, *P* < 0.001) and longer hospital stay (74.0 vs 50.0 days, *P* < 0.001, Table 3).

**Table 3:** The primary and secondary outcomes of infants by infection status

Outcomes	Ureaplasma associated pneumonia (*n* = 56)	Ureaplasma colonization (*n* = 62)	*p*-Value
BPD, n (%)	25 (44.6)	11 (17.7)	0.002
Hospital death, n (%)	4 (7.1)	1 (1.6)	0.189
Effective azithromycin treatment^a^, n (%)	18 (32.1)	-	-
ROP, n (%)	17 (30.4)	12 (19.4)	0.166
Duration of invasive ventilation, days, median (IQR)	1 (8)	0 (1)	0.004
Duration of non-invasive ventilation, days, median (IQR)	37 (41)	19.5 (30)	< 0.001
Duration supplemental oxygen, days, median (IQR)	58 (44)	33 (30)	< 0.001
Duration of hospitalization, days, median (IQR)	74 (45)	50 (26)	< 0.001

*Abbreviations*: *BPD* Bronchopulmonary dysplasia, *IQR* Interquartile range, *ROP* Retinopathy of prematurity

^a^data calculated in infants defined as Ureaplasma associated pneumonia only

We further analysed the association between Ureaplasma-associated pneumonia and BPD, as well as the association between effective azithromycin treatment and BPD, in the multivariate logistic analysis. Univariate analysis was shown in supplemental Table 1. Although a higher rate of Ureaplasma-associated pneumonia was observed in BPD infants compared with those without BPD (58.6% vs. 24.7%), the association did not reach statistically significant after adjustment [OR 1.835, 95%CI (0.548, 6.147), Table 4]. An effective azithromycin treatment was significantly associated with a reduced risk for BPD [OR 0.011, 95% CI (0.000, 0.250), Table 4].

**Table 4:** Multivariate logistic analysis on Ureaplasma associated variates and BPD

Variates	BPD (*n* = 36)	Non-BPD (*n* = 82)	Adjusted OR (95% CI)	*p*-Value
Gestational age, mean (SD)	26.4 (1.5)	28.8(1.7)	0.939 (0.485, 1.819)	0.852
Birth Weight, mean (SD)	899 (187)	1237 (262)	0.995 (0.990, 1.000)	0.066
Male, n (%)	27 (75)	45 (54.9)	3.842 (1.132, 13.038)	0.031
1 min Apgar Score (IQR)	8 (5)	9 (2)	1.073 (0.743, 1.549)	0.706
5 min Apgar Score (IQR)	10 (1)	10(0)	0.765 (0.326, 1.797)	0.539
Surfactant, n (%)	31 (86.1)	34 (41.5)	1.593 (0.388, 6.543)	0.518
Intubation, n (%)	28 (77.8)	19 (23.2)	3.485 (1.002, 12.117)	0.050
Ureaplasma associated pneumonia before treatment, n (%)	17 (58.6)	22 (24.7)	1.835 (0.548, 6.147)	0.325
Effective azithromycin treatment, n (%)^a^	1 (4.1)	17 (54.8)	0.011 (0.000, 0.250)	0.005

*Abbreviations BPD* Bronchopulmonary dysplasia, *IQR* Interquartile range. All included variates for multivariate logistical analysis were displayed in the table

^a^calculated in infants defined as Ureaplasma associated pneumonia only (*N* = 56, of which 18 infants defined as effective azithromycin treatment) and adjusted for gestational age, birth weight, gender, Apgar^1−min^, Apgar^5−min^, surfactant and intubation

## Discussion

This study showed that Ureaplasma-associated pneumonia before treatment was not independently associated with BPD development in Ureaplasma positive VLBW infants, despite a remarkably higher rate of BPD in infants with Ureaplasma-associated pneumonia compared to those with colonization (44.6% vs. 17.7%). In contrast, an effective azithromycin treatment was significantly associated with decreased risk for BPD in Ureaplasma positive VLBW infants [OR 0.011, 95% CI (0.000, 0.250)].

Ureaplasma is smaller than bacteria in diameter and colonizes in the genitourinary tract of pregnant women. Ureaplasma might transmit to the new-borns respiratory tract in the uterus or during delivery, causing interstitial pneumonia and closely associated BPD in premature infants [8]. Some speculations are proposed on the role of Ureaplasma in the pathogenesis of BPD. Phospholipase released by Ureaplasma has long been considered as the virulence factor causing epithelium necrosis [7]. Infants who died from Ureaplasma pneumonia exhibited severe fibrosis, indicating fibroblast involvement [21]. Besides, Ureaplasma infection increased the infiltration of inflammatory cells, releasing cytokines of interleukin 6 (IL-6), matrix metalloproteinase 8 (MMP8), tumour necrosis factor *α* (TNF*α*) and contributing to late-onset sepsis (LOS) [14, 21]. These pathophysiological processes justify using interstitial pneumonia as an essential parameter for Ureaplasma infection in the current study. A recent meta-analysis demonstrated a persistent association between Ureaplasma and BPD in preterm infants. However, our findings suggest that an infective pattern of Ureaplasma was not independently associated with the onset of BPD.

Since Ureaplasma in broncho alveolar lavage fluids was first reported in 1988 [22], the role of Ureaplasma in morbidities of prematurity, especially in BPD, has drawn the great interest of neonatologists [5, 9, 23, 24]. However, infants tested positive for Ureaplasma are not routinely treated in NICUs, given that no evidence supports that eradication of Ureaplasma reduces the risk for BPD [12]. Several researchers speculated that the pattern of Ureaplasma infection might determine its contribution to BPD. Castro-Alcaraz et al. reported that only persistent Ureaplasma colonization was associated with BPD [10]. Maternal Ureaplasma abundance might increase the risk of BPD [25]. In the current study, we found Ureaplasma-associated pneumonia was not significantly associated with a higher risk of BPD, despite a striking clinical effect being noticed.

An effective azithromycin treatment was significantly associated with a reduced risk of BPD. These sort of contradictory findings indicate that azithromycin treatment might work by other mechanisms than eradicating Ureaplasma, including but not strict to the anti-inflammatory effect of azithromycin [26]. Viscardi et al. had previously reported that administration of azithromycin at a daily dose of 20 mg/kg for three days effectively eradicated Ureaplasma from the respiratory tract and tended to reduce BPD. However, the effect was not statistically significant, which could be attributed to the under power of the study [12]. The same authors reported an increased rate of death or serious respiratory morbidities in Ureaplasma positive infants (58%) compared to negative infants (21%) in a 2-years-follow-up study of the trial, indicating a compromised lung function in Ureaplasma positive infants [27].

We acknowledge that the clinical setting regarding Ureaplasma, azithromycin and BPD is highly complicated. The complicated issues include the association between Ureaplasma and BPD, azithromycin and pneumonia resolution, the influence of pneumonia on BPD, the effect of azithromycin on the development of BPD, etc. Addressing these issues would be challenging even in a series of well-designed prospective studies. However, we hope the findings in our study could add to the body of evidence regarding these topics. There could be some confounders compromising the accuracy of CXR assessment. A severe RDS might interfere with the initial assessment of CXR (20% in this cohort), while other concurrent pulmonary infections and PDA might impede the second accurate assessment of CXR. Furthermore, sampling from tracheal aspirates would be more accurate than nasopharyngeal swabs in PCR testing for Ureaplasma [27].

In summary, we found that radiographic improvement following azithromycin treatment was associated with a reduced risk for BPD in Ureaplasma positive VLBW infants. However, these data need to be interpreted with caution since potential confounders might affect the accuracy of the CXR evaluation.

## Supplementary Information

**Additional file 1: Supplemental Figure.** Representative photos of CXR in diffuse granularity, interstitial changesand emphysema, with none, mild and severe changes.

**Additional file 2: Supplemental Table 1.** Clinical characteristics stratified by BPD status.

## Data Availability

The raw data supporting the conclusions of this article will be made available by the corresponding authors to any qualified researcher.
