# Supplementary figures and images for: Association of Ureaplasma infection pattern and azithromycin treatment effect with bronchopulmonary dysplasia in Ureaplasma positive infants: a cohort study
Source: BMC Pulm Med. 2023 Jun 26;23:229. doi: 10.1186/s12890-023-02522-4 (PMC10294412; doi:10.1186/s12890-023-02522-4)

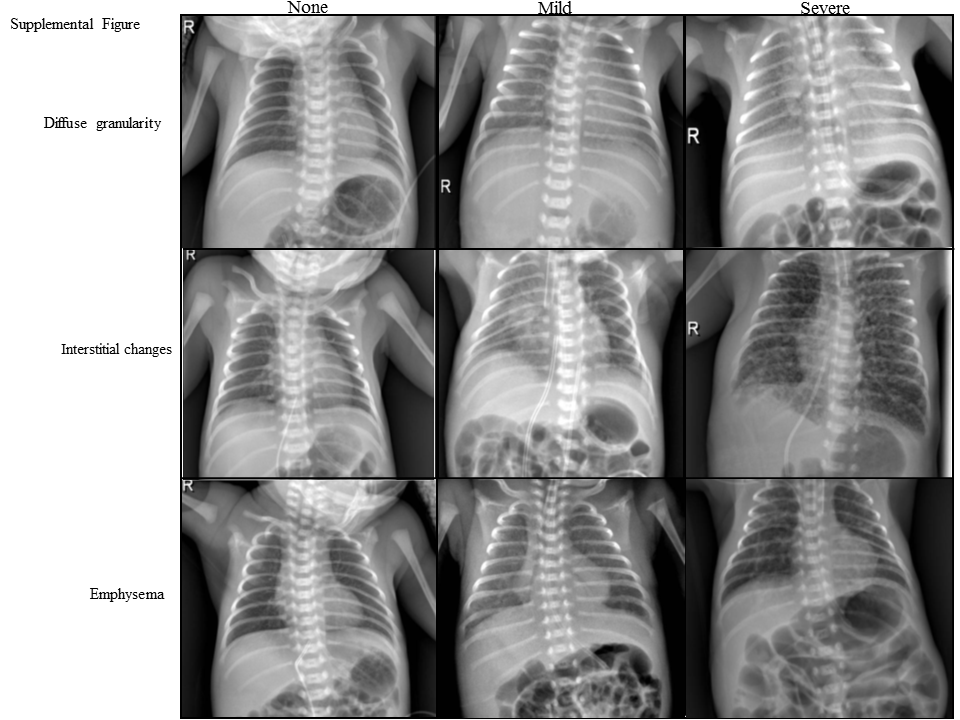

Supplement: Supplementary file 1 — Additional file 1: Supplemental Figure. Representative photos of CXR in diffuse granularity, interstitial changesand emphysema, with none, mild and severe changes. [file 12890_2023_2522_MOESM1_ESM.tif]
