# Supplementary material for: Association of Ureaplasma infection pattern and azithromycin treatment effect with bronchopulmonary dysplasia in Ureaplasma positive infants: a cohort study
Source: BMC Pulm Med. 2023 Jun 26;23:229. doi: 10.1186/s12890-023-02522-4 (PMC10294412; doi:10.1186/s12890-023-02522-4)
Supplement: Supplementary file 2 — Additional file 2: Supplemental Table 1. Clinical characteristics stratified by BPD status. [file 12890_2023_2522_MOESM2_ESM.docx]

Supplemental Table 1. Clinical characteristics stratified by BPD status.

| Characteristic | BPD | Non-BPD | P value |
| --- | --- | --- | --- |
|  | (n=36) | (n=82) |  |
| Male, n (%) | 27 (75) | 45 (54.9) | 0.039 |
| GA, mean (SD) | 26.4 (1.5) | 28.8(1.7) | <0.001 |
| BW, mean (SD) | 899 (187) | 1237 (262) | <0.001 |
| Gestational hypertension, n (%) | 0 (0) | 1 (1.6) | 1.000 |
| Gestational diabetes, n (%) | 5 (13.9) | 16 (19.5) | 0.462 |
| Vaginal delivery, n (%) | 24 (66.7) | 51 (62.2) | 0.642 |
| 1 min Apgar Score, median (IQR) | 8 (5) | 9 (2) | 0.003 |
| 5 min Apgar Score, median (IQR) | 10 (1) | 10(0) | 0.023 |
| Antenatal steroid, n (%) | 27 (75) | 69 (84) | 0.240 |
| NRDS (grade II and above), n (%) | 7 (19.4) | 17 (20.7) | 0.873 |
| SGA, n (%) | 2 (5.6) | 5 (6.1) | 0.909 |
| hsPDA, n (%) | 13 (36.1) | 19 (23.2) | 0.145 |
| PPROM, n (%) | 19 (52.8) | 51 (62.2) | 0.338 |
| Surfactant use, n (%) | 31 (86.1) | 34 (41.5) | 0.000 |
| Intubation, n (%) | 28 (77.8) | 19 (23.2) | 0.000 |
| EOS, n (%) | 5 (13.9) | 8 (9.8) | 0.509 |
| Ureaplasma associated pneumonia before treatment, n (%) | 17 (58.6) | 22 (24.7) | 0.002 |
| Effective azithromycin treatment^a^, n/n (%) | 1/25 (4) | 17/31 (54.8) | <0.001 |

Abbreviations: GA, gestational age; SD, standard deviation; BW, Birth weight; IQR, interquartile range; NRDS, neonatal respiratory distress syndrome; SGA, small for gestational age; hsPDA, hemodynamically significant patent ductus arteriosus; PPROM, prelabor rupture of the membrane; EOS, early-onset sepsis, including suspected and culture proven. a, data calculated in infants defined as Ureaplasma associated pneumonia before treatment (25/56 infants with BPD).
